# Intermittent Preventive Treatment for Malaria in Papua New Guinean Infants Exposed to *Plasmodium falciparum* and *P. vivax*: A Randomized Controlled Trial

**DOI:** 10.1371/journal.pmed.1001195

**Published:** 2012-03-27

**Authors:** Nicolas Senn, Patricia Rarau, Danielle I. Stanisic, Leanne Robinson, Céline Barnadas, Doris Manong, Mary Salib, Jonah Iga, Nandao Tarongka, Serej Ley, Anna Rosanas-Urgell, John J. Aponte, Peter A. Zimmerman, James G. Beeson, Louis Schofield, Peter Siba, Stephen J. Rogerson, John C. Reeder, Ivo Mueller

**Affiliations:** 1 Papua New Guinea Institute of Medical Research, Madang, Papua New Guinea; 2 Department of Medicine, University of Melbourne, Melbourne Australia; 3 Swiss Tropical and Public Health Institute, Basel, Switzerland; 4 University of Basel, Basel, Switzerland; 5 Infection and Immunity Division, Walter and Eliza Hall Institute, Melbourne, Australia; 6 Barcelona Centre for International Health Research (CRESIB), Barcelona, Spain; 7 Center for Global Health and Diseases, Case Western Reserve University, Cleveland, Ohio, United States of America; 8 Burnet Institute, Melbourne, Australia; St George's Hospital Medical School, United Kingdom

## Abstract

**Background:**

Intermittent preventive treatment in infants (IPTi) has been shown in randomized trials to reduce malaria-related morbidity in African infants living in areas of high *Plasmodium falciparum (Pf)* transmission. It remains unclear whether IPTi is an appropriate prevention strategy in non-African settings or those co-endemic for *P. vivax (Pv)*.

**Methods and Findings:**

In this study, 1,121 Papua New Guinean infants were enrolled into a three-arm placebo-controlled randomized trial and assigned to sulfadoxine-pyrimethamine (SP) (25 mg/kg and 1.25 mg/kg) plus amodiaquine (AQ) (10 mg/kg, 3 d, *n* = 374), SP plus artesunate (AS) (4 mg/kg, 3 d, *n* = 374), or placebo (*n* = 373), given at 3, 6, 9 and 12 mo. Both participants and study teams were blinded to treatment allocation. The primary end point was protective efficacy (PE) against all episodes of clinical malaria from 3 to 15 mo of age. Analysis was by modified intention to treat. The PE (compared to placebo) against clinical malaria episodes (caused by all species) was 29% (95% CI, 10–43, *p*≤0.001) in children receiving SP-AQ and 12% (95% CI, −11 to 30, *p* = 0.12) in those receiving SP-AS. Efficacy was higher against *Pf* than *Pv*. In the SP-AQ group, *Pf* incidence was 35% (95% CI, 9–54, *p* = 0.012) and *Pv* incidence was 23% (95% CI, 0–41, *p* = 0.048) lower than in the placebo group. IPTi with SP-AS protected only against *Pf* episodes (PE = 31%, 95% CI, 4–51, *p* = 0.027), not against *Pv* episodes (PE = 6%, 95% CI, −24 to 26, *p* = 0.759). Number of observed adverse events/serious adverse events did not differ between treatment arms (*p*>0.55). None of the serious adverse events were thought to be treatment-related, and the vomiting rate was low in both treatment groups (1.4%–2.0%). No rebound in malaria morbidity was observed for 6 mo following the intervention.

**Conclusions:**

IPTi using a long half-life drug combination is efficacious for the prevention of malaria and anemia in infants living in a region highly endemic for both *Pf* and *Pv*.

**Trial registration:**

ClinicalTrials.gov NCT00285662

*Please see later in the article for the Editors' Summary*

## Abstract 2

A three-arm randomized trial conducted among infants in Papua New Guinea estimates the preventive effect against malaria episodes of intermittent preventive treatment, in an area where children are exposed to both falciparum and vivax malaria.

## Editors' Summary

**Background:**

Malaria is a major global public health problem. Half the world's population is at risk of this parasitic disease, which kills about one million people (mainly young children in sub-Saharan Africa) every year. Most of these deaths are caused by *Plasmodium falciparum* but *P. vivax*, the commonest and most widely distributed malaria parasite, is a major cause of malaria-related morbidity (illness and death) in many of the tropical and subtropical regions of the world where malaria is endemic (always present). Malaria is transmitted to people through the bites of night-flying mosquitoes. It can be prevented by controlling the mosquitoes that spread the parasite and by sleeping under insecticide-treated nets to avoid mosquito bites. Prompt treatment of malaria with antimalarial drugs can also reduce malaria transmission. In addition, intermittent preventative treatment (IPT)—the treatment of symptom-free individuals with full therapeutic courses of antimalarial drugs at fixed intervals regardless of their infection status—has been shown to reduce malaria-related morbidity among pregnant women in malaria-endemic areas and among African infants living in areas of high *P. falciparum* transmission.

**Why Was This Study Done?:**

The World Health Organization recently recommended that, in Africa, IPT should be given during infancy (called IPTi) at the same time as routine immunizations. Because the studies on which this recommendation is based were all carried out in sub-Saharan Africa, in populations where *P. falciparum* is the predominant parasite and *P. vivax* is uncommon, it is not known whether IPTi would be an appropriate prevention strategy in non-African settings or in regions where both *P. falciparum* and *P. vivax* are endemic. In this randomized placebo-controlled trial, the researchers investigate the efficacy of IPTi in infants living in an area of Papua New Guinea where *P. falciparum* and *P. vivax* are both highly endemic. In a randomized placebo-controlled trial, the effects of an intervention and of a placebo (dummy) intervention are compared in groups of individuals chosen through the play of chance.

**What Did the Researchers Do and Find?:**

The researchers assigned more than 1,000 infants to receive sulfadoxine/pyrimethamine (SP) plus amodiaquine (AQ) (SP and AQ are long-lasting antimalarial drugs), SP plus artesunate (AS) (AS is a short-lasting antimalarial), or placebo at 3, 6, 9, and 12 months old. They recorded the number of malaria episodes that occurred among the children between the ages of 3 and 15 months. Then, by comparing the number of episodes occurring among the children receiving SP-AS or SP-AQ with the number occurring among the children receiving placebo, the researchers calculated the protective efficacy of the two drug combinations over the study period. The protective efficacy of IPTi against all clinical malaria episodes (*P. falciparum* and *P. vivax* combined) was 29% for SP-AQ, but SP-AS was not associated with a statistically significant reduction in all malaria episodes as compared to placebo. For the two species of malaria separately, the incidence of *P. falciparum* malaria was 35% lower among the children receiving SP-AQ than among the children receiving placebo, whereas the incidence of *P. vivax* was reduced by 23%; IPTi with SP-AS provided protection only against *P. falciparum* malaria (protective efficacy 31%). Importantly, the number of adverse events (possible drug side effects) was similar in all the treatment arms, none of the severe adverse events were treatment-related, and there was no rebound in malaria-related morbidity for six months following the end of the intervention.

**What Do These Findings Mean?:**

These findings show that IPTi using a combination of long-lasting antimalarial drugs (SP-AQ) can effectively and safely prevent malaria in a non-African population living in a region where *P. falciparum* and *P. vivax* are both highly endemic. Importantly, they also show that IPTi with SP-AQ can prevent both *P. falciparum* and *P. vivax* malaria. For Papua New Guinea, these findings suggest that SP-AQ is an appropriate drug choice for IPTi, particularly since the replacement of SP-AQ by artemether-lumefantrine as the national first line treatment for malaria will reduce the selection pressure for resistance against SP and AQ. However, although these finding provide proof-of-principle evidence for the efficacy and safety of IPTi, further studies are needed to identify the most effective combinations of long-lasting antimalarial drugs for use in IPTi in other malaria-endemic regions.

**Additional Information:**

Please access these web sites via the online version of this summary at http://dx.doi.org/10.1371/journal.pmed.1001195.

- Information is available from the World Health Organization on malaria (in several languages); the 2011 World Malaria Report provides details of the current global malaria situation; and the WHO policy recommendation on IPTi for P. falciparum malaria control in Africa is available
- The US Centers for Disease Control and Prevention provide information on malaria (in English and Spanish), including a selection of personal stories about malaria
- Information is available from the Roll Back Malaria Partnership on the global control of malaria, including a fact sheet about malaria in children and information on malaria in Papua New Guinea
- The IPTi Consortium was established to evaluate IPTi and inform public health policy making
- The Malaria Vaccine Initiative has a fact sheet on P. vivax malaria
- Vivaxmalaria.com provides information about *P. vivax*
- MedlinePlus provides links to additional information on malaria (in English and Spanish)

## Introduction

Malaria and anemia are major causes of morbidity and mortality in children in tropical countries [1]. Several preventive strategies have proven to be effective in reducing this burden, such as insecticide-treated bed nets [2], indoor residual spraying [3], or prompt diagnosis and treatment of malaria with artemisinin combination therapy [1],[4]. Additionally, the preventative administration of a full treatment course of antimalarial drugs at fixed intervals regardless of illness episodes, named intermittent preventive treatment (IPT), has been shown to reduce malaria-related morbidity in different high risk groups [5],[6]. This strategy is increasingly widely used in pregnant women (IPTp) [7],[8], with a positive effect on antenatal parasitemia, placental malaria, maternal hemoglobin (Hb), birth weights, and newborn malaria infections [7],[9]. Several studies have shown that IPT is effective in reducing malaria incidence when given to infants at the time of routine immunizations (IPTi), or to older children in areas where malaria transmission is highly seasonal (IPTc) [10]–[13].

In the past 10 y, eight randomized controlled trials have investigated IPTi in different African countries, with various drug regimens. Sulfadoxine-pyrimethamine (SP), dispensed as a single dose three or four times during the first year of life, is the most studied drug and has been associated with a protective efficacy (PE) against clinical malaria episodes of 30% (ranging from 20% to 59%) in populations where SP resistance is not extensive. It also reduced the risk of anemia by 21% in these trials [10]. In all but one trial the preventative effect of the IPTi dose was restricted almost exclusively to the first 5 wk after an individual IPTi dose, with little effect thereafter [10]. While IPTi with SP retains efficacy in areas with moderate SP resistance [14],[15], SP has failed to prevent malaria in areas with very high resistance levels [16],[17]. Based on this evidence, the World Health Organization recommends IPTi as a suitable malaria control intervention in areas of high malaria burden and low-to-moderate SP resistance [18].

To date, all reported IPTi studies have been carried out exclusively in sub-Saharan Africa in populations where *Plasmodium falciparum (Pf)* is the predominant parasite and *P. vivax (Pv)* is uncommon because of the high prevalence of Duffy negativity [19]. However, in highly endemic areas of the Southwest Pacific [20],[21], Southeast Asia [22], the Americas [23], and the horn of Africa [24], *Pv* is a major cause of malaria morbidity, including severe illness and death in young children in particular. Therefore, although IPTi may have significant benefits in these regions, to date there are no data of which we are aware on IPTi efficacy in non-African settings, or on the use of IPTi for malaria due to *P. vivax*. Results from Africa cannot be easily extrapolated to other settings because of differences in the biology of *Pf* and *Pv* [25], in particular the ability of *Pv* to relapse from long-lasting liver stages, and also the ability of *Pv* to quickly acquire resistance to SP [26].

In order to determine the efficacy of IPTi in reducing the burden of malaria (all cause as well as *Pf* and *Pv* specific) and anemia in a *Pf* and *Pv* co-endemic setting, we undertook a three-arm randomized placebo-controlled trial of IPTi in an area of Papua New Guinea (PNG) that is highly endemic for both *Pf* and *Pv*. We tested two different drug regimens, combining a long-acting drug (SP) with either 3 d of amodiaquine (AQ) (another long half-life drug) or 3 d of artesunate (AS) (a short half-life drug), given in conjunction with routine immunization activities. These two drug regimens were chosen for two reasons: (1) they were the first and second line treatments in PNG at the time of the commencement of the study, facilitating their possible implementation into PNG standard practice as treatments for IPTi, and (2) the comparison allows differentiation between the importance of potentiallyimproved clearance of existing infections (using SP-AS) versus better prevention of new infections post-treatment (using the long-acting combination of SP-AQ).

## Methods

### Trial Design, Sites, and Study Participants

We undertook an individually randomized placebo-controlled trial of two different drug regimens (SP [single dose] in combination with either 3 d of AQ [SP-AQ] or 3 d of AS [SP-AS]) given as IPTi at routine vaccination time points (3, 6, and 9 mo) and at 12 mo in conjunction with routine vitamin A supplementation as part of the World Health Organization's Expanded Programme on Immunization (EPI) in PNG. Participants, field teams, and investigators were all blinded with respect to treatment allocation. The efficacy of the intervention was assessed for a 12-mo period from enrollment to a primary assessment time point at 15 mo of age, with a 6-mo extended follow-up (15–21 mo) to assess potential rebound.

The study was carried out between 6 June 2006 and 14 May 2010 on the north coast of PNG in the Mugil area of Sumkar District, Madang Province. The study site included 20 villages situated in an ∼400-km^2^ coastal area 30–60 km north of Madang town. The region receives over 3,000 mm of rainfall annually, with a short dry season (June to October), and is considered to have hyper-endemic malaria [27],[28]. Study villages are serviced by two major health centers (Mugil and Alexishafen) and several aid posts that are responsible for delivering EPI through monthly outreach clinics. Although ownership of bed nets was relatively high (>50%), until the start of free long-lasting insecticide-treated net distribution by the national malaria program in late 2008, the majority of the population in the study site were using untreated nets.

Originally, the trial was started as a two-site study, with a second site in Wosera (East Sepik Province). However, an interim analysis performed mid-study found that the incidence of malaria was dramatically different between the two sites, restricting the comparability of the two sites. Consequently, enrollments were ceased in Wosera in May 2008 and, in line with a revised sample size calculation, enrollments at the Madang site were increased to 334 children per arm. The data from the Wosera cohort were subsequently used only for an overall safety analysis (Table S1).

The study was carried out in accordance with Good Clinical Practice (ICH GCP E6) guidelines and externally monitored by two independent monitors and the Data Safety Monitoring Board. The study was approved by the PNG Medical Research Advisory Committee (MRAC number 05.20). The trial was registered on http://www.clinicaltrials.gov (number NCT00285662) and formed part of the IPTi Consortium (http://www.ipti-malaria.org) [29].

### Randomization, Blinding, and Treatment Allocation

Children were enrolled if they fulfilled the following criteria: (1) permanent resident of the area, (2) aged 3 (±1) mo old, (3) no disability, (4) no chronic illness, (5) no known allergy to study drugs, (6) Hb>5 g/dl, and (7) no severe malnutrition (defined by the PNG national guidelines as a weight less than 60% of the Harvard reference weight-for-age value [30]). After enrollment children were randomly allocated to the placebo, SP-AQ, or SP-AS treatment group using a pre-assigned list. Randomization lists were prepared by independent statistician using SAS software and consisted of assignments in blocks of 12, each comprising a list of the same 12 letters in random order. The three arms of the trial were each assigned four randomly selected letters from among the twelve, which were used to label the packaging of the tablets. The code list was held by the statistician in a locked drawer until completion of the trial. Individual allocation codes were contained in pre-prepared opaque envelopes, which were opened only after a child was allocated to the next available ID number.

SP, AS, AQ, and their respective placebos were pre-packaged in individual blisters by the drug manufacturers (SP and AS, IPCA; AQ, Kinapharma). Study drugs and placebo were identical in appearance and color, but as drugs were administered with sweet syrup no attempt was made to match the moderately bitter taste of AQ. The quality of all drugs and corresponding placebo was independently verified. Study drugs and placebo were assigned to the respective treatment codes by an independent technician. The entire study team and principal investigators remained fully blinded for the entire study period.

### Clinical Study Procedures

The three different study interventions were (1) SP-AQ, a single dose of SP (25 mg/kg sulfadoxine and 1.25 mg/kg pyrimethamine) combined with 3 d of AQ (10 mg/kg) plus 3 d of AS placebo; (2) SP-AS, a single dose of SP plus 3 d of AS (4 mg/kg) plus 3 d of AQ placebo; (3) matching placebos for all three drugs. The tablets were split (quarters or halves) according to the weight (in kilograms) of each child. The two treatment arms corresponded to the first and second line treatments of the PNG national standard guidelines for malaria at that time.

The intervention was delivered four times during the first year of life alongside the EPI at 3, 6, 9, and 12 mo of age (Table 1). The drugs were crushed and mixed with water and sweet syrup (Golden Crush Cordial, Coca-Cola Amatil) for easy administration with spoon or syringe. The first day dose was administered by study staff. The children were monitored closely, and if they vomited within 30 min of receiving treatment, the dose was repeated. The second and third doses were given by the carer at home without direct supervision. Adherence and potential adverse reactions to the study drugs were assessed by community reporters, and an adherence rate of >90% was reported (data not shown).

**Table 1:** Summary of scheduled patient contacts during study follow-up.

Type of Visit	Age (Months)							
	2	3	6	9	12	15	18	21
Pre-screening	X							
IPTi treatment		IPTi 1	IPTi 2	IPTi 3	IPTi 4			
Primary efficacy assessment						X		
Follow-up							X	
Final assessment								X
Passive case detection		X	X	X	X	X	X	X
Immunization	OPV 3	OPV 4						
		HBV 3						
	DPT 2	DPT 3						
			Meas 1	Meas 2				
			Vit A		Vit A			
During schedules visits								
Blood collection (finger prick)		500 µl	250 µl	250 µl	250 µl	1 ml	250 µl	250 µl
Hb levels		X				X		

DTP, diphteria, pertussis, tetanus vaccine; HBV, hepatitis B vaccine; Meas, measles vaccine; OPV, oral polio vaccine; Vit A, oral vitamin A supplementation.

The parents or guardians of potential study participants were contacted during monthly routine EPI clinics run jointly by study staff and health center staff when the child was 1–2 mo of age. The study was explained in detail to the parents through both individual and community awareness meetings. An information brochure and consent form in English and in the local language (Pidgin) was given to the parents to take home for further discussion.

A child who met the inclusion criteria was formally enrolled during the next clinic visit, when the child was 3 mo of age (±1 mo) and after at least one parent gave written consent. Upon enrollment, a concise medical history including bed net use, possible disabilities, and presence of acute illness was performed. A brief physical examination was done, including measurement of weight. Table 1 summarizes what occurred at different visits during the trial.

Throughout the study period, a passive case detection system was maintained at the Mugil health center and three outlying clinics where study participants received treatment free of charge. Each illness episode was assessed by study staff using a standard case report form. In the case of history of fever within the past 48 h or an axillary temperature >37.5°C, a rapid malaria diagnostic test (ICT Combo) was performed, two blood smears and 250 ml of whole blood were collected, and Hb level was measured using a portable HemoCue 201 machine. Only infants positive for malaria on the rapid diagnostic test were treated with artemether/lumefantrine (Coartem). The children with moderate to severe anemia (Hb<8 g/dl) received iron supplementation (ferrous sulfate, 5 mg/kg daily for 6 wk). All other illnesses were treated according to the standard treatment guidelines of PNG. Children presenting with any danger signs or symptoms were referred to the health center for admission and treatment. Study participants who attended after hours were treated by health center staff based on presumptive diagnosis. In such cases, study staff recorded the reason for admission and (when possible) performed a finger prick to check parasitemia and Hb the next day.

All illness episodes were considered adverse events (AEs) and were graded by the study clinicians according to severity from grade 1 (less severe) to grade 3 or as a serious adverse event (SAE; life-threatening or deadly, resulting in disability/incapacity, or presenting with specific clinical/laboratory features such as those that characterize important hematological disorders [as defined by the IPTi Consortium]). Hospital admissions that did not fulfill the criteria of SAE were considered grade 3. The study clinicians assessed all SAEs and causality of AEs. SAEs and AEs possibly or certainly related to the intervention were reported to the Data Safety Monitoring Board, which assessed grade and causality using IPTi Consortium guidelines.

### Diagnosis of Malaria Infections

All blood slides were read by two expert microscopists. In case of discrepancies, a third read was performed. Thick blood films were examined by light microscopy for 200 thick-film fields (under 100× oil immersion lens) before being declared infection-negative. Slides were scored as light-microscopy-positive for an individual *Plasmodium* species, if the species was detected independently by at least two microscopists. Additionally, if the final slide readings were discrepant compared to PCR results (positivity and species), a final confirmatory read by a senior microscopist was performed. Parasite densities were recorded as the number of parasites per 200 white blood cells and converted to the number of parasites/microliter assuming 8,000 white blood cells/microliter [3]. Final parasite densities were obtained by calculating the geometric mean of positive reads.

Blood samples were collected from finger pricks in tubes coated with ethylenediaminetetraacetic acid and/or in plain tubes (15-mo visit only). Upon arrival in the laboratory, samples were separated into plasma or serum and cell pellet. Plasma and serum were stored at −80°C until further use. DNA was extracted from cell pellets using the QiaAMP 96 extraction kit (Qiagen) and the 96-well Genomic DNA Extraction Kit (Favorgen).

The presence of each of the four human malarial species was assessed in all blood samples using a semi-quantitative post-PCR ligase detection reaction/fluorescent microsphere assay (LDR-FMA) [31]. This assay combines PCR amplification of the small subunit ribosomal RNA gene (491- to 500-bp fragments) using genus-specific primers, followed by a multiplex species-specific ligation detection reaction. The design and sensitivity of this assay have been described previously [31],[32]. The threshold of positivity of the reactions for each species was determined monthly using the mean value obtained for the negative controls (for each species), plus three times the value of its standard deviation. Samples with low-positive values (<[threshold+8× standard deviation]) were double-checked and considered as positive only if the result was confirmed.

### Sample Size Calculations

Based on a predicted incidence rate of 1.2 episodes/child/year (0.7 for *Pf* and 0.5 for *Pv*), a power of 90% and alpha = 0.025, a sample size of 250 children per arm was estimated to be necessary to find at least a 28% reduction in the incidence of all malaria episodes, a 30% reduction in the incidence of *Pv*, and a 35%–37% reduction in the incidence of *Pf* episodes. Assuming a drop out of participants of 20% in conjunction with a fall in incidence of malaria of 30% to 50% due to long-lasting insecticide-treated net introduction through the national program in November 2008, a final adjusted sample size of 366 children per arm, or 1,100 children in total, was calculated.

### Statistical Methods

The analysis was performed both by modified intention to treat (mITT) and according to protocol (ATP). The mITT population includes all randomized children in Mugil that received at least one dose of IPTi or placebo (i.e., 1,121 of 1,125 children). Following the mITT principle, participants were analyzed according to the preventive treatment they were assigned to at randomization. In the ATP analysis, the population includes all randomized participants who received at least three IPTi treatments (each given within 4 wk after the scheduled treatment time point) and were under passive surveillance for the entire 12-mo period.

The primary objective of the trial was to evaluate the protective effect of the two IPTi regimens. The primary outcome was measured as the incidence of symptomatic malaria due to any *Plasmodium* species from 3 to 15 mo of age. Secondary outcomes included (1) the specific incidence of symptomatic malaria due to *Pf* or *Pv* from 3 to 15 mo of age, (2) the incidence of symptomatic malaria (any, *Pf*, and *Pv*) from 15 to 21 mo (i.e., “rebound”), (3) the incidence of moderate-to-severe anemia (Hb<8 g/dl) and severe anemia (Hb<5 g/dl) from 3 to 15 mo and 15 to 21 mo, and (4) the prevalence of malaria parasitemia at 15 and 21 mo.

Symptomatic malaria was defined as history of fever or axillary temperature ≥37.5°C and a positive blood smear or a positive rapid diagnostic test, confirmed by positive PCR. The incidences were compared between groups using negative binomial regression to take into account possible extra-Poisson variation due to frailty at the individual level. Analyses were adjusted by sex, number of IPTi treatments received, season of enrollment (wet versus dry), average bed net use, and site (grouped by 12 recruitment zones). The time at risk was calculated starting on the date of enrollment until the date defined according to the analysis, or until the participant was withdrawn from the study. An arbitrary period of 28 d was excluded after each malaria episode. The same methodology was used for evaluating of the incidence of moderate-to-severe (Hb<8 g/dl) and severe anemia (Hb<5 g/dl). Efficacy results are presented as either incidence rate ratio (IRR) or PE (PE = 1−IRR).

The risk of malaria within 35 d following the administration of each of the treatment doses was investigated by calculating incidence rates and hazard ratios using a Cox regression model. Differences in prevalence of infection at 15 and 21 mo were evaluated using logistic regression.

The analyses were performed in Stata version 11 (StataCorp).

## Results

**Figure 1:**
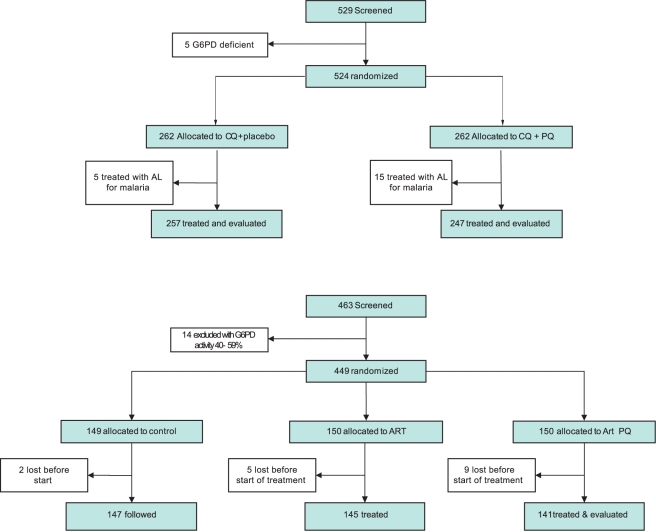
Trial profile. incomplete FU, incomplete follow-up (i.e., the study was terminated before the participant completed the 21-mo follow-up time period); LTF, lost to follow-up.

From 6 June 2006 to 14 May 2010, 1,125 3-mo-old infants were enrolled, randomized, and followed-up in the trial in the Mugil area of Madang Province (Figure 1). Four infants were retrospectively excluded from the analysis as they were already receiving antimalarial treatments at the time of all IPTi visits and therefore did not receive any IPTi treatment doses. In all, 374 infants were allocated to the SP-AQ group, 374 to the SP-AS group, and 373 to the placebo group (Figure 1). A total of 1,079 (96%) participants completed follow-up to 15 mo, whereas 857 (76%) completed the 6-mo post-treatment follow-up (i.e., 15–21 mo).

The baseline characteristics of all study infants were similar across the three treatment arms (Table 2). No significant imbalances were observed between the three groups. In the final multivariate models, gender, village of residence, average insecticide-treated net use, and season of recruitment were included. A total of 939 children who received three or four IPTi treatments were included in the ATP population, with no significant imbalances between groups (Table S2).

**Table 2:** Baseline characteristics of study participants—intention-to-treat cohort.

Characteristic	Subcategory	Placebo	SP-AQ	SP-AS
**Total ** ***n***		373	374	374
**Female**		192 (51.5%)	166 (44.4%)	187 (50%)
**Village area**	Biranis	18 (4.8%)	20 (5.4%)	18 (4.8%)
	Megiar	20 (5.4%)	17 (4.6%)	22 (5.9%)
	Aronis Garup Wasabamal Zizzi	41 (10%)	35 (9.4%)	42 (11.2%)
	Basken Dimer	77 (20.6%)	74 (19.9%)	72 (19.3%)
	Bunu Kusen Mugil	38 (10.2%)	54 (14.4%)	54 (14.4%)
	Matukar Wasab	38 (10.2%)	31 (8.3%)	24 (6.4%)
	Dylup	20 (5.4%)	29 (7.8%)	22 (5.9%)
	Karkum	16 (4.3%)	8 (2.1%)	20 (5.6%)
	Mirap	17 (4.6%)	23 (6.2%)	31 (8.3%)
	Sareng	30 (8%)	21 (5.6%)	23 (6.2%)
	Taldig	41 (11%)	48 (12.8%)	33 (8.8%)
	Rempi	17 (4.6%)	14 (3.7%)	13 (3.5%)
**Slept under bed net last 2 wk** a		308 (82.8%)	299 (80%)	305 (82%)
**Recruitment during rainy season (October–June)** a		303 (73.5%)	301 (73.0%)	301 (72.5%)
**Mean age at enrollment (days)** a		96 (15)	99 (18)	98 (16)
**Weight (kilograms)** a		5.9 (0.8)	6.0 (0.9)	6.0 (0.8)
**Hb (grams/decaliter)** a		9.5 (1.0)	9.5 (1.2)	9.5 (1.1)
**Prevalence of parasitemia at baseline (light microscopy)**	All species	14 (3.8%)	30 (8%)	24 (6.4%)
	*Pf*	7 (1.9%)	13 (3.5%)	9 (2.4%)
	*Pv*	7 (1.9%)	17 (4.5%)	15 (4%)
**Prevalence of parasitemia at baseline (PCR)**	All species	69 (18.5%)	87 (23.3%)	75 (20.1%)
	*Pf*	17 (4.6%)	20 (5.4%)	15 (4%)
	*Pv*	52 (13.9%)	74 (19.8%)	63 (16.8%)

Data are *n* (percent) unless otherwise indicated.

a Data are mean (standard deviation).

### Protective Efficacy

The incidence of clinical malaria between the first dose at 3 mo and 15 mo in the placebo arm was 1.07 per person-year at risk (PYAR) overall, and 0.28 and 0.82 for *Pf* and *Pv*, respectively. In mITT analyses, the incidence of all cases of malaria was 29% (95% CI, 10–43, *p*≤0.001) lower in the SP-AQ group as compared to placebo, but not significantly reduced in the SP-AS group as compared to placebo (point estimate for change 12%, 95% CI, −11 to 30, *p* = 0.12). The PE was higher against *Pf* than *Pv* in both groups (Table 3). In the SP-AQ group, *Pf* incidence was reduced by 35% (95% CI, 9–54, *p* = 0.012) and *Pv* incidence by 23% (95% CI, 0–41, *p* = 0.048) in comparison to placebo. In contrast, the SP-AS group showed a significant reduction only for incidence of *Pf* (PE = 31%, 95% CI, 4–51, *p* = 0.027), not for incidence of *Pv* (PE = 4%, 95% CI, −26 to 24, *p* = 0.759).

**Table 3:** Efficacy of IPTi with SP-AQ and SP-AS against malaria and anemia, overall as well as after and between individual doses.

Analysis	End Points	Placebo			SP-AQ					SP-AS					*p*-Value^a^
		Events	PYAR	Incidence	Events	PYAR	Incidence	IRR	95% CI	Events	PYAR	Incidence	IRR	95% CI	
**Efficacy results of IPTi 3–15 mo—mITT cohort**	**Clinical malaria**														
	All episodes	362	337.3	1.07	280	352.1	0.80	0.71	0.57–0.90	336	346.9	0.97	0.88	0.70–1.11	0.018
	All *Pf* episodes	100	356.1	0.28	67	366.6	0.18	0.65	0.46–0.91	71	365.4	0.19	0.69	0.49–0.96	0.020
	*Pf*>2,500/µl	70	358.0	0.20	31	369.0	0.08	0.43	0.28–0.66	47	367.2	0.13	0.65	0.45–0.96	<0.001
	All *Pv* episodes	282	342.9	0.82	234	355.4	0.66	0.77	0.59–1.00	283	350.5	0.81	0.96	0.74–1.24	0.108
	*Pv*>500/µl	219	346.8	0.63	180	358.8	0.50	0.75	0.56–1.01	227	354.2	0.64	0.99	0.74–1.32	0.111
	**Severe malaria**	12	362.1	0.03	10	370.4	0.03	0.81	0.33–1.97	11	369.6	0.03	0.89	0.37–2.13	0.894
	**Anemia**														
	Hb<8 g/dl	169	351.1	0.48	132	362.6	0.36	0.75	0.56–1.01	133	361.2	0.37	0.75	0.56–1.01	0.090
	Hb<5 g/dl	8	362.4	0.02	2	371.1	0.01	0.24	0.05–1.15	4	370.2	0.01	0.49	0.15–1.63	0.125
	**All cause mortality**	6	362.9	0.017	0	371.2	0	—	—	1	370.4	0.03	—	—	0.011
**Efficacy results of IPTi 3–15 mo—ATP cohort**	**Clinical malaria**														
	All episodes	302	285.8	1.06	212	310.5	0.68	0.62	0.48–0.80	250	300.0	0.83	0.77	0.60–0.99	0.001
	All *Pf* episodes	83	301.6	0.28	45	321.7	0.14	0.51	0.35–0.74	47	314.1	0.15	0.54	0.38–0.78	<0.001
	*Pf*>2,500/ml	57	303.3	0.19	21	323.2	0.06	0.35	0.21–0.57	29	315.5	0.09	0.49	0.31–0.77	<0.001
	All *Pv* episodes	235	290.4	0.81	180	312.8	0.58	0.69	0.52–0.91	218	302.3	0.72	0.88	0.66–1.16	0.033
	*Pv*>500/ml	180	312.8	0.58	137	315.4	0.43	0.70	0.50–0.97	173	305.2	0.57	0.93	0.68–1.28	0.075
	**Severe malaria**	11	306.4	0.036	7	324.2	0.022	0.60	0.22–1.66	9	316.8	0.028	0.79	0.30–2.05	0.612
	**Anemia**														
	Hb<8 g/dl	143	297.2	0.48	92	318.9	0.29	0.59	0.42–0.82	101	310.4	0.33	0.66	0.47–0.92	0.004
	Hb<5 g/dl	7	306.7	0.023	1	324.7	0.003	0.13	0.02–1.10	2	317.4	0.006	0.28	0.06–1.33	0.043
**Incidence of malaria in the 35 d following and in between treatment doses**	**Prophylactic effect**														
	All episodes	101	104.4	0.97	23	114.7	0.20	0.20	0.12–0.33	39	108.3	0.36	0.37	0.24–0.55	<0.001
	All *Pf* episodes	27	107.5	0.25	8	115.2	0.07	0.28	0.13–0.67	5	109.6	0.05	0.18	0.07–0.47	<0.001
	All *Pv* episodes	82	105.2	0.78	16	115.1	0.17	0.17	0.10–0.30	35	108.4	0.41	0.41	0.27–0.63	<0.001
	**Inter-dose effect**														
	All episodes	202	170.7	1.18	168	183.2	0.92	0.77	0.58–1.01	197	172.6	1.14	0.96	0.74–1.25	0.118
	All *Pf* episodes	56	178.0	0.31	34	189.5	0.18	0.57	0.37–0.87	34	180.1	0.19	0.62	0.41–0.94	0.016
	All *Pv* episodes	154	173.2	0.89	145	184.3	0.79	0.89	0.66–1.21	172	173.8	0.99	1.13	0.84–1.52	0.305

a *p*-Values for comparison across all three treatment groups.

Estimates of protection were higher when only children with three or more IPTi treatments were considered (i.e., ATP analyses; Table 3). Regular administration of SP-AQ reduced malaria episodes by 38% overall (95% CI, 20–52, *p*<0.001), with reductions of 49% (95% CI, 26–65, *p*<0.001) and 31% (95% CI, 9–48, *p* = 0.010) for *Pf* and *Pv* malaria episodes, respectively. Administration of SP-AS resulted in a 23% reduction in all malaria episodes (95% CI, 1–40, *p* = 0.041), with a statistically significant reduction for *Pf* (46% lower, 95% CI, 22–62, *p* = 0.001) but not for *Pv* (point estimate for change 12%, 95% CI, −16 to 34, *p* = 0.355).

Comparable effects were seen when more specific definitions for *Pf* (i.e., parasite density >2,500/µl) and *Pv* (i.e., parasite density >500/µl) [33] were applied (Table 3). In the ATP cohort, in the SP-AQ arm only, a substantially higher protection was observed against higher density *Pf* (PE = 65%, 95% CI, 43–79, *p*<0.001) but not against higher density *Pv*. The results for the mITT cohort are comparable.

IPTi with SP-AQ or SP-AS also provided protection against moderate-to-severe anemia (Hb<8 g/dl; Table 3). In the ATP population, the PE increased to 41% (95% CI, 18–58, *p* = 0.002) in the SP-AQ group and 34% (95% CI, 8–53, *p* = 0.013) in the SP-AS group. Few cases of severe anemia (<5 g/dl) were observed during the study. In the ATP analysis, there were seven cases in the placebo group, one in the SP-AQ group, and two in the SP-AS group (*p* = 0.04). Results for the mITT population were of similar magnitude, although in general the estimates obtained were lower and not statistically significant (*p* = 0.09–0.13) (see Table 3).

**Figure 2:**
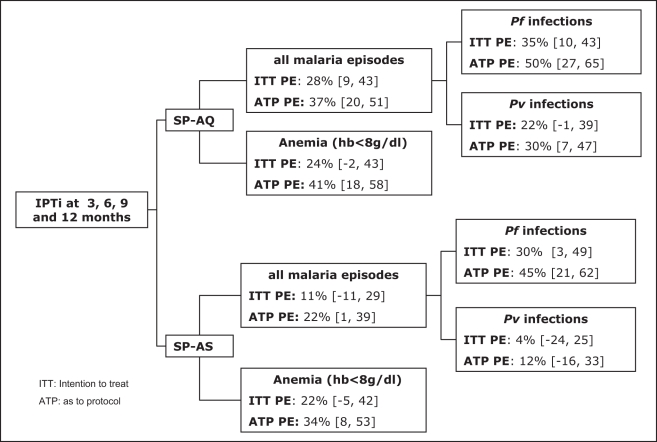
Summary of IPTi preventive efficacy against malaria at 15 mo of age adjusted for sex, place of residence, season of enrollment, and average insecticide-treated net use.

Adjustment for gender, village of residency, season of recruitment, and use of bed nets did not significantly alter estimates of PE (Figure 2).

### Post- and Inter-Dose Efficacy

The combined PE in the 35 d following the administration of each IPTi treatment dose showed a reduction of 80% (95% CI, 67–88, *p*<0.001) with SP-AQ and 63% (95% CI, 45–76, *p*<0.001) with SP-AS for all malaria infection (Table 3). SP-AQ had slightly lower PE against *Pf* (72%, 95% CI, 33–87, *p*<0.001) than against *Pv* (83%, 95% CI, 70–90, *p*<0.001), whereas SP-AS was more efficacious against *Pf* (82%, 95% CI, 53–93, *p*<0.001) than against *Pv* (59%, 95% CI, 47–73, *p*<0.001). Both SP-AQ (PE = 43%, 95% CI, 13–63, *p* = 0.010) and SP-AS (PE = 38%, 95% CI, 6–59, *p* = 0.026) were associated with a significant reduction in the incidence of *Pf* malaria between IPTi doses (i.e., the period from 35 d after a treatment dose to the next IPTi dose). However, no effect on the incidence of *Pv* malaria was observed for either treatment group beyond the first 35 d (Table 3).

### Safety

A total of 6,165 AEs and 275 SAEs were observed during the intervention period, with no significant difference between treatment arms (Table 4). None of the SAEs were judged (by the Data Safety Monitoring Board and IPTi Safety Working Group) to be intervention-related. One child in the placebo group experienced a non-severe (grade 1) skin rash a few days following administration of the second IPTi dose. Vomiting was reported in 2% of infants receiving SP-AQ, 1.4% of infants receiving SP-AS, and 0.6% of infants receiving placebo (*p* = 0.001).

**Table 4:** Safety of IPTi with SP-AQ and SP-AS: adverse events and effect of incidence of malaria and anemia.

Analyses and Endpoints	Placebo			SP-AQ					SP-AS					*p*-Value^b^
	Events	PYAR	Incidence	Events	PYAR	Incidence	IRR^a^	95% CI	Events	PYAR	Incidence	IRR^a^	95% CI	
**Safety of IPTi 3–15 mo - mITT cohort**														
All adverse events	1,353	359.4	3.76	1,348	367.7	3.67	0.97	0.88–1.08	1,353	366.8	3.69	0.98	0.88–1.09	0.867
Serious adverse events	65	362.8	0.18	83	371.0	0.22	1.22	0.83–1.79	81	370.1	0.22	1.20	0.81–1.77	0.550
**Clinical malaria**														
All episodes	171	149.0	1.15	164	155.5	1.05	0.91	0.70–1.20	167	157.2	1.06	0.92	0.70–1.21	0.763
All Pf episodes	65	156.2	0.42	68	162.0	0.42	1	0.68–1.48	74	163.8	0.45	1.08	0.74–1.58	0.907
Pf>2500/µl	41	157.8	0.26	43	163.6	0.26	1.01	0.63–1.62	51	165.0	0.31	1.19	0.76–1.88	0.693
All Pv episodes	115	152.9	0.75	112	158.8	0.71	0.93	0.67–1.30	106	161.1	0.66	0.88	0.63–1.23	0.753
Pv>500/µl	86	154.9	0.56	84	160.6	0.52	0.93	0.63–1.38	78	162.9	0.48	0.87	0.58–1.29	0.779
**Anemia**														
Moderate-to-severe (Hb<8 g/dl)	42	157.7	0.27	44	163.4	0.27	1.01	0.63–1.61	45	165.4	0.27	1.03	0.64–1.63	0.994
**Clinical malaria**														
All episodes	140	127.4	1.10	138	136,2	1.01	0.91	0.67–1.24	143	134.6	1.06	0.96	0.71–1.30	0.833
All Pf episodes	54	133.1	0.41	56	141.8	0.39	0.97	0.63–1.48	63	140.1	0.45	1.10	0.73–1.67	0.809
Pf>2500/µl	34	134.5	0.25	35	143.2	0.24	0.96	0.57–1.62	44	141.2	0.31	1.25	0.75–2.03	0.560
All Pv episodes	94	130.6	0.72	95	138.9	0.68	0.95	0.66–1.37	91	137.9	0.66	0.92	0.64–1.34	0.911
Pv>500/µl	71	132.2	0.54	72	140.5	0.51	0.94	0.61–1.46	68	139.4	0.49	0.92	0.59–1.43	0.928
**Anemia**														
Moderate-to-severe (Hb<8 g/dl)	40	134.1	0.30	37	142.9	0.26	0.87	0.43–1.44	39	141.6	0.28	0.93	0.56–1.43	0.862

a Model-based IRR.

b *p*-Values for comparison across all three treatment groups.

In total, ten deaths occurred among study participants. One was excluded from the analysis because he/she did not receive any IPTi treatment dose. Out of the nine study deaths, seven occurred during the intervention period (i.e., 3–15 mo of age). Of these, six occurred in the placebo group, one in the SP-AS group, and none in the SP-AQ group (*p* = 0.011). An additional two deaths (one each in the SP-AS and placebo groups) were observed during post-intervention follow-up. The likely diagnoses for the nine deaths were as follows: one severe *Pv* malaria (parasite density of 12,200/µl) with severe anemia and possible lower respiratory tract infection (severe respiratory distress and cough), three lower respiratory tract infections, two severe dehydrations, one meningitis, and two unknown diagnoses.

### Post-Intervention Risk

The incidence of clinical malaria between 15 and 21 mo was 1.09 PYAR overall and 0.43 and 0.70 for *Pf* and *Pv*, respectively. No evidence of a “rebound effect” was observed in the 6 mo following the end of the intervention at the age of 15 mo. In both mITT and ATP populations, no significant difference in incidence of all malaria episodes was observed between treatment and intervention groups (Table 4; *p* = 0.56). Similarly, no significant differences in incidence of *Pf* malaria, *Pv* malaria, and anemia were observed.

### Prevalence of Parasitemia during Follow-Up

**Figure 3:**
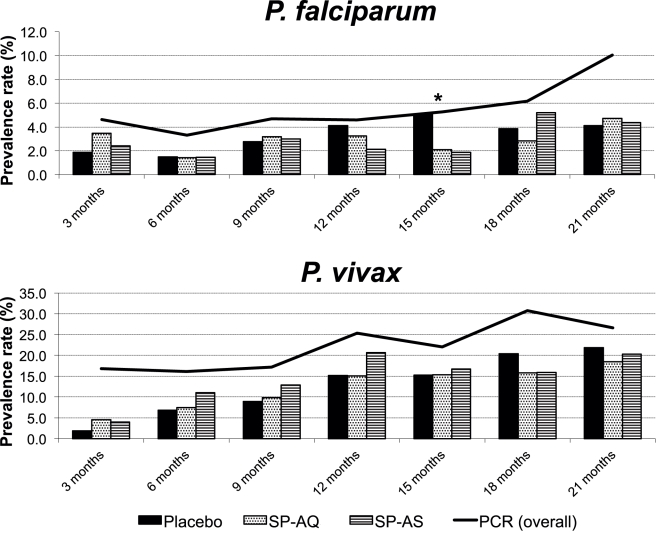
Prevalence of *P. falciparum* and *P. vivax* infections during follow-up by treatment group (light microscopy) and overall (PCR). * *p*<0.05.

The prevalence rates of both *Pf* and *Pv* increased from 1.5% and 8.5% at 6 mo to 3.0% and 15.8% at 15 mo and 4.4% and 20.3% at 21 mo, respectively, when detected by microscopy (Figure 3). Using molecular diagnosis, prevalence of *Pf* and *Pv* was higher, and also increased with age (Figure 3). At 15 mo, the prevalence of *Pf* was significantly lower in the two treatment arms (*p* = 0.037) by light microscopy but not by LDR-FMA. No significant differences in prevalence were observed between treatment arms at 21 mo or any other time point for either *Pf* or *Pv*. Infections with *P. malariae* and *P. ovale* were rare by microscopy (overall prevalence *P. malariae*: 0.1%, *P. ovale*: 0.0%) and LDR-FMA (*P. malariae*: 1.0%, *P. ovale*: 0.4%).

## Discussion

This study in PNG infants provides the first evidence of which we are aware for the efficacy of IPTi with SP-based regimens for the prevention of malaria and anemia in a non-African population, including data on the efficacy against *Pv* malaria. IPTi with a combination of SP plus 3 d of AQ resulted in a 29% decrease in all malaria episodes and a 25% decrease in episodes of moderate-to-severe anemia. PE was higher in children who received at least three doses of IPTi (38% for all malaria and 41% for anemia). In line with local resistance patterns and SP-AS pharmacokinetic properties, SP-AS was found to be inferior to SP-AQ. Overall, the efficacy of IPTi with SP-AQ in PNG infants is comparable to the 30% efficacy observed in other studies of African infants receiving IPTi with SP [10]. Importantly, SP-AQ has significant efficacy against both *Pf* and *Pv* malaria. Both IPTi treatments were safe and well tolerated. Therefore, these findings provide essential proof-of-principle evidence for the safety and efficacy of IPTi outside Africa, which has important implications for public health policy in non-African settings.

### Prevention of Malaria

Overall, IPTi with SP-AQ was more effective in preventing *Pf* than *Pv* malaria (mITT 35% versus 23%, ATP 49% versuss 31%), and was more effective than SP-AS for *Pf* and *Pv* malaria. This was not unexpected. Two recent African clinical trials of IPTi have investigated alternative drug regimens, including at least one long half-life antimalarial such as mefloquine (125 mg single dose), a combination of SP (single dose) plus 3 d of AS, or AQ plus AS. These treatments had protective efficacies ranging from 26% to 38% [16],[34] and were comparable to those in earlier SP trials. However, a shorter acting combination, dapsone/chlorproguanil failed to significantly reduce the burden of malaria or anemia [16],[34]. Similarly, in the earlier IPTi SP trials, the preventative effect of IPTi SP was almost entirely due to a reduction of malaria in the first 5 wk after treatment [10],[35]. Together, these results suggest that IPTi works mainly through a prophylactic effect, achieved by long-acting drugs preventing new infections, rather than by eradicating existing parasitemia [35]. Consistent with the African results, in the present study both SP-containing regimens had similarly high protective effect against *Pf* for the first 35 d (72%–82%) as well as a moderate inter-dose effect, resulting in an overall efficacy of IPTi against *Pf* that was towards the higher end of those found in African studies [10],[16],[34].

SP-AQ also prevented 83% of *Pv* episodes in the first 35 d following treatment, indicating a similar post-treatment prophylactic effect, but the overall efficacy of IPTi against *Pv* malaria was lower than for *Pf*. SP-AS, however, showed significantly less post-treatment prophylactic effect (PE = 59%) and no effect against *Pv* malaria overall. The most probable reasons for this are the ability of *Pv* to relapse from long-lasting liver stages [36] and higher levels of SP resistance in *Pv*. *Pv* strains from New Guinea are thought to relapse very frequently [37], and *Pv* blood-stage infections are therefore re-established soon after drug treatment. In a concurrently conducted drug trial, 49% of PNG children 0.5–5 y treated with SP-chloroquine (3 d) for *Pv* malaria had recurrent *Pv* parasitemia, and an additional 18% had a recurrent *Pv* clinical episode by day 42, while 26% and 6% of children treated for *Pf* malaria had recurrent *Pf* parasitemia and clinical episodes, respectively [38].

The differential efficacy of IPTi with SP-AQ and SP-AS for the prevention of *Pf* and *Pv* malaria is consistent with the pharmacokinetic properties of the drugs used and previously observed drug resistance patterns. PCR-corrected day 28 in vivo failure rates from studies conducted concurrently or just prior to the trial were 10.3% for SP-AQ [39] and 10.0% for SP-AS [38] for *Pf*, and 3.6% for SP-AQ [40] and 11.8% for SP-AS [41] for *Pv*. As SP has never been used as monotherapy in PNG, there are no estimates of SP in vivo resistance for either *Pf* or *Pv*. However, molecular markers of SP resistance have been analyzed for both species in the study population. In *Pf*, the quintuple *Pf dihydrofolate reductase (pfdhfr)* 51–59–108/*Pf dihydropteroate synthase (pfdhps)* 436–547 mutant genotype is associated with a high degree of drug resistance [42], while in *Pv*, the 57L-58R-61M-117T *pvdhfr* quadruple mutation (irrespective of *pvdhps* mutations) has been associated with increased risk of treatment failure [43],[44]. The high frequency of the quadruple *pvdhfr* mutation in PNG (52% [41]) suggests that the clinical efficacy of SP for *Pv* may be significantly compromised. In contrast, the absence of quadruple/quintuple *pfdhfr*/*pfdhps* [45] suggests that SP retains substantial efficacy against *Pf*.

Although chloroquine-resistant *Pv* was first described in PNG [46], there is a significantly higher level of 4-aminoquinoline resistance present in *Pf* than in *Pv*. By the early 1990s, greater than 35% of *Pf* cases showed RII and RIII resistance [47], and mutations underlying chloroquine and AQ resistance were shown to be highly prevalent [48]. In 2003/2004, SP-AQ was 100% clinically effective, but recurrent *Pv* parasitemia was observed in 12% of children treated with SP-AQ [39]. The *pvdhfr* 57L-58R-61M-117T/*Pv* multidrug resistance 1 *(pvmdr1)* 976F quintuple mutant genotype has been associated with parasitological failure [43]. Although the *pvmdr1* 976F mutation was present in ∼70% of *Pv* isolates tested in 2006/2007, the predominantly late occurrence of recurrent parasitemia (i.e., day 28–42) following treatment with SP-chloroquine indicates that 4-aminoquinolines retained good clinical and at least partial parasitological efficacy [41].

Given the high level of AQ resistance and the very short half-life of AS [49], the efficacy of IPTi for the prevention of *Pf* is likely to be largely due to the effect of SP. The high level of preventative efficacy for 35 d is consistent with the terminal elimination half-life (*t*_1/2β_) of 15.6 and 9.1 d for pyrimethamine and sulfadoxine, respectively, in PNG infants [50]. With SP having a greatly reduced efficacy against *Pv*, the success of IPTi against *Pv* malaria relies largely on the efficacy of the partner drug. Whereas *Pv* parasites are efficiently cleared by SP-AS, the short half-life of AS provides no post-treatment prophylactic effect, and recurrent *Pv* infections are very common [38]. AQ on the other hand has a half-life of ∼9 d [51], and a 3-d course should therefore effectively suppress sensitive parasites effectively for 4–5 wk. The lack of any protective effect of SP-AS against *Pv* but not *Pf* adds further evidence to the observation that while SP can retain a considerable effect at low-to-moderate levels of resistance [14], it loses its prophylactic activity at high levels of resistance [16].

### Prevention of Anemia, Severe Illness, and Death

Besides reducing the burden of malaria episodes, IPTi also reduced the risk of moderate-to-severe anemia (25% in the mITT and 34%–41% in the ATP analyses, respectively) and severe anemia (51%–87%). These effects are in line with previous findings on the prevention of anemia in Africa [10].

No difference was observed between the groups in number of SAEs (i.e., all cause admissions plus severe illness). Surprisingly, a significantly lower death rate was observed in the two treatment arms compared to the placebo arm. Even though this finding is very encouraging, it must be interpreted with caution. The present study was not powered to observe an effect of IPTi on mortality, and in the meta-analysis of six African studies, no effect of IPTi with SP on mortality was detected [10]. Only seven deaths occurred during the intervention period, with most children dying at home. Based on verbal autopsies and available clinical records, a majority of children might have died of severe lower respiratory tract infections and not from malaria.

### Safety of SP-AQ and SP-AS

IPTi with SP-AQ or SP-AS was very safe. More than 4,000 doses of SP and 6,000 doses of AQ and AS were given, and no study-drug-related SAEs were observed, while AEs such as vomiting were rare in all groups. As reported in earlier studies, no significant increase in risk of malaria or anemia was recorded in the 6 mo after completion of the 12 mo of IPTi, indicating that IPTi does not impair the acquisition of immunity to both *Pf* and *Pv* and lead to a rebound in malaria risk. These data are very reassuring in regard to a possible use of these drug combinations in PNG and add to the safety data from African IPTi studies [10].

The use of SP as prophylaxis has been criticized because of the potential risk of Stevens-Johnson syndrome, a severe skin reaction [52], but by now >15,000 children have been treated with SP-containing IPTi regimens in well-controlled clinical trials, and not a single drug-related severe dermatological event has been observed [10],[16],[34],[53]. On the other hand, IPTi with mefloquine was highly effective, but was not well tolerated, with high rates of vomiting [16]. In the absence of novel drug regimens for IPTi, the combination of SP and AQ is the best currently available option for IPTi in PNG.

### Adherence, Acceptability, and Implementation

The size of the difference in IPTi efficacy between the ATP and mITT analyses indicates that if the intervention is implemented, it will be important to achieve high coverage and high adherence in order to achieve good effectiveness.

In both the SP-AQ and SP-AS groups only the first dose was given as directly observed treatment. Parents were then counseled on the importance of giving the remaining two doses at home. With this approach a very high rate of compliance (>90%) was achieved. As a result, SP and AQ day 4 drug levels were found to be comparable (N. Senn, P. Rarau, and I. Mueller, unpublished data) to those found in pharmacological studies with directly observed drug administration [50],[54]. Acceptability of IPTi by parents and health workers of IPTi was excellent, and the administration of IPTi alongside EPI did not have negative impacts on attitudes to EPI, EPI adherence, or existing malaria prevention practices [55]. Therefore, and consistent with what was described in Africa [53], the IPTi intervention seems to be well suited for implementation in community settings in PNG, with high likelihood of both acceptability and adherence.

### Conclusion

This study provides evidence of the efficacy of IPTi for the prevention of malaria and anemia in a region highly endemic for both *Pf* and *Pv*. Additionally, this study provides an essential proof of principle that IPTi is an appropriate strategy for the prevention of *Pv* malaria, if an effective, long half-life drug is used. Policy makers should therefore consider IPTi in areas outside Africa or in countries in the Horn of Africa, where the burden of *Pv* malaria is high. Which areas are suited for IPTi introduction will depend on transmission level and choice of drug, both of which will affect the cost-effectiveness of the intervention [56]. While the results from Africa indicate that IPTi is a highly cost-effective intervention even at moderate levels of transmission [57], a formal evaluation of IPTi cost-effectiveness in non-African settings will be required to assist policy makers. Given the levels of resistance to SP and AQ in many parts of the Asia-Pacific and Americas, further studies are needed to investigate other combinations of long-acting drugs with a better efficacy against *Pv*, in particular.

In the PNG context, the combination of SP-AQ (two long-acting drugs with well-matched half-lives and good activity against either *Pf* [SP] or *Pv* [AQ]) is an appropriate drug choice for IPTi, and its introduction into the national standard treatment guidelines should be considered. The replacement of AQ and SP by artemether-lumefantrine as the national first line treatment will reduce the selection pressure for resistance against AQ and SP. Therefore, the efficacy of the two drugs in IPTi may be retained over time. Appropriate monitoring of prophylactic efficacy of AQ and SP, as well as evaluating new regimens, such as dihydroartemisinin-piperaquine, should accompany IPTi introduction in PNG.

## Supporting Information

Table S1 Safety of IPTi with SP-AQ and SP-AS: AEs from both Madang and Maprik cohorts.
(DOCX)

Table S2 Baseline characteristics of study participants—ATP cohort.
(DOCX)

## Abbreviations

AE: adverse event
AQ: amodiaquine
AS: artesunate
ATP: according to protocol
EPI: Expanded Programme on Immunization
Hb: hemoglobin
IPT: intermittent preventive treatment
IPTi: intermittent preventive treatment in infants
IRR: incidence rate ratio
LDR-FMA: ligase detection reaction/fluorescent microsphere assay
mITT: modified intention to treat
PE: protective efficacy
*Pf*: *Plasmodium falciparum*
PNG: Papua New Guinea
*Pv*: *Plasmodium vivax*
PYAR: person-year at risk
SAE: serious adverse event
SP: sulfadoxine-pyrimethamine
