# Correction: Intermittent Preventive Treatment for Malaria in Papua New Guinean Infants Exposed to *Plasmodium falciparum* and *P. vivax*: A Randomized Controlled Trial

**DOI:** 10.1371/annotation/de06fdd3-c263-416c-8b5f-291f9c474558

**Published:** 2012-06-07

**Authors:** Nicolas Senn, Patricia Rarau, Danielle I. Stanisic, Leanne Robinson, Céline Barnadas, Doris Manong, Mary Salib, Jonah Iga, Nandao Tarongka, Serej Ley, Anna Rosanas-Urgell, John J. Aponte, Peter A. Zimmerman, James G. Beeson, Louis Schofield, Peter Siba, Stephen J. Rogerson, John C. Reeder, Ivo Mueller

The corresponding author provided an incorrect trial profile as Figure 1 due to an oversight during the copyediting process. Please view the correct version of Figure 1 here: 

**Figure:**
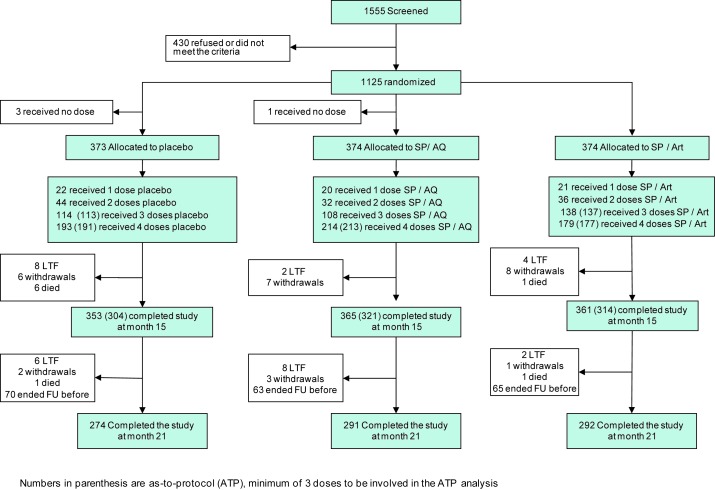


. The legend for Figure 1 is correct as published.

