# Supplementary material for: Intermittent Preventive Treatment for Malaria in Papua New Guinean Infants Exposed to Plasmodium falciparum and P. vivax: A Randomized Controlled Trial
Source: PLoS Med. 2012 Mar 27;9(3):e1001195. doi: 10.1371/journal.pmed.1001195 (PMC3313928; doi:10.1371/journal.pmed.1001195)
Supplement: Table S1 — Safety of IPTi with SP-AQ and SP-AS: AEs from both Madang and Maprik cohorts. (DOCX) [file pmed.1001195.s001.docx]

**Supplemental Table 1**: Safety of IPTi with SP-AQ and SP-AS: Adverse Events from both Madang and Maprik cohorts

|  | **Placebo** | |  |  | **SP-AQ** |  |  |  |  |  | **SP-AS** |  |  |  |  |  |  |
| --- | --- | --- | --- | --- | --- | --- | --- | --- | --- | --- | --- | --- | --- | --- | --- | --- | --- |
|  | **Events** | **PYAR^1^** | **Incidence** |  | **Events** | **PYAR^1^** | **Incidence** | **IRR^2^** | **95% CI** |  | **Events** | **PYAR^1^** | **Incidence** | **IRR^2^** | **95% CI** |  | **p-value^3^** |
|  |  |  |  |  |  |  |  |  |  |  |  |  |  |  |  |  |  |
| **Safety of IPTi 3-15 months - mITT cohorts** | | | |  |  |  |  |  |  |  |  |  |  |  |  |  |  |
|  |  |  |  |  |  |  |  |  |  |  |  |  |  |  |  |  |  |
| All adverse events | 2035 | 512.1 | 3.97 |  | 2078 | 519.5 | 4.00 | 1.00 | 0.91-1.09 |  | 2052 | 518.3 | 3.96 | 1.00 | 0.91-1.08 |  | 0.848 |
|  |  |  |  |  |  |  |  |  |  |  |  |  |  |  |  |  |  |
| Serious adverse events | 82 | 517.1 | 0.16 |  | 99 | 524.7 | 0.19 | 1.11 | 0.78-1.56 |  | 94 | 523.5 | 0.18 | 1.07 | 0.76-1.52 |  | 0.328 |
|  |  |  |  |  |  |  |  |  |  |  |  |  |  |  |  |  |  |

^1^PYAR: Person-year at risk; ^2^IRR: Incidence rate ratio (model based); ^3^p – values for comparison across all 3 treatment groups.
