# Supplementary material for: Intermittent Preventive Treatment for Malaria in Papua New Guinean Infants Exposed to Plasmodium falciparum and P. vivax: A Randomized Controlled Trial
Source: PLoS Med. 2012 Mar 27;9(3):e1001195. doi: 10.1371/journal.pmed.1001195 (PMC3313928; doi:10.1371/journal.pmed.1001195)
Supplement: Table S2 — Baseline characteristics of study participants—ATP cohort. (DOCX) [file pmed.1001195.s002.docx]

| **Supplemental Table 2: Baseline characteristics of study participants – According-to-protocol (ATP) cohort** | | | | |
| --- | --- | --- | --- | --- |
|  | | **PLACEBO** | **SP-AQ** | **SP-AS** |
| N |  | 304 | 321 | 314 |
| Girls |  | 156 (51.3%) | 138 (43.0%) | 157 (50.0%) |
| Village areas | Biranis | 16 (5.3%) | 18 (5.6%) | 16 (5.1%) |
|  | Megiar | 14 (4.6%) | 16 (5.0%) | 19 (6.1%) |
|  | Aronis Garup Wasabamal Zizzi | 33 (10.9%) | 31 (9.7%) | 38 (12.1%) |
|  | Basken Dimer | 72 (23.7%) | 63 (19.6%) | 59 (18.8%) |
|  | Bunu Kusen Mugil | 30 (9.9%) | 46 (14.37%) | 45 (14.3%) |
|  | Matukar Wasab | 29 (9.5%) | 27 (8.47%) | 20 (6.4%) |
|  | Dylup | 19 (6.3%) | 26 (8.1%) | 16 (5.1%) |
|  | Karkum | 12 (4.0%) | 8 (2.5%) | 18 (5.74%) |
|  | Mirap | 15 (4.9%) | 17 (5.3%) | 25 (8.00%) |
|  | Sareng | 18 (5.9%) | 19 (5.9%) | 18 (5.7%) |
|  | Taldig CCI | 30 (9.9%) | 38 (11.8%) | 29 (9.2%) |
|  | Rempi | 16 (5.3%) | 12 (3.7%) | 11 (3.5%) |
| Slept under bednet last two weeks | | 249 (82.2%) | 256 (79.8%) | 262 (83.7%) |
| Recruitment during rainy season (September-June) | | 250 (82.2%) | 255 (79.4%) | 257 (81.9%) |
| Mean Age at enrollment (days) | | 6.0 (0.8) | 6.0 (0.9) | 6.0 (0.8) |
| Weight (Kg) |  | 5.9 ( 0.8) | 6.0 ( 0.9) | 6.0 ( 0.8) |
| Hemoglobin (g/dl) |  | 9.6 (1.0) | 9.5 (1.2) | 9.6 (1.0) |
| Prevalence of parasitemia at baseline (light microscopy) | All species | 11 (3.6%) | 24 (7.5%) | 18 (5.7%) |
|  | Pf | 5 (1.6%) | 11 (3.4%) | 7 (2.2%) |
|  | Pv | 6 (2.0%) | 13 (4.1%) | 11 (3.5%) |
| Prevalence of parasitemia at baseline (PCR) | All species | 55 (18.15) | 70 (21.8%) | 61 (19.4%) |
|  | Pf | 15 (4.9%) | 14 (4.4%) | 12 (3.8%) |
|  | Pv | 41 (13.5%) | 61 (19.0%) | 54 (17.2%) |
| Data are means (SD) and n(%) | |  |  |  |
| ** wild-type |  |  |  |  |
